# Disparity in the quality of COVID-19 data reporting across India

**DOI:** 10.1186/s12889-021-11054-7

**Published:** 2021-06-24

**Authors:** Varun Vasudevan, Abeynaya Gnanasekaran, Varsha Sankar, Siddarth A. Vasudevan, James Zou

**Affiliations:** 1 Institute for Computational & Mathematical Engineering, Stanford University, Palo Alto, California USA; 2 Independent Researcher, Palo Alto, California USA; 3 Independent Researcher, Neuchatel, Switzerland; 4 Department of Biomedical Data Science, Stanford University, Palo Alto, California, USA

**Keywords:** Data reporting quality framework, Pandemic data reporting, COVID-19, India, Coronavirus

## Abstract

**Background:**

Transparent and accessible reporting of COVID-19 data is critical for public health efforts. Each Indian state has its own mechanism for reporting COVID-19 data, and the quality of their reporting has not been systematically evaluated. We present a comprehensive assessment of the quality of COVID-19 data reporting done by the Indian state governments between 19 May and 1 June, 2020.

**Methods:**

We designed a semi-quantitative framework with 45 indicators to assess the quality of COVID-19 data reporting. The framework captures four key aspects of public health data reporting – availability, accessibility, granularity, and privacy. We used this framework to calculate a COVID-19 Data Reporting Score (CDRS, ranging from 0–1) for each state.

**Results:**

Our results indicate a large disparity in the quality of COVID-19 data reporting across India. CDRS varies from 0.61 (good) in Karnataka to 0.0 (poor) in Bihar and Uttar Pradesh, with a median value of 0.26. Ten states do not report data stratified by age, gender, comorbidities or districts. Only ten states provide trend graphics for COVID-19 data. In addition, we identify that Punjab and Chandigarh compromised the privacy of individuals under quarantine by publicly releasing their personally identifiable information. The CDRS is positively associated with the state’s sustainable development index for good health and well-being (Pearson correlation: *r*=0.630,*p*=0.0003).

**Conclusions:**

Our assessment informs the public health efforts in India and serves as a guideline for pandemic data reporting. The disparity in CDRS highlights three important findings at the national, state, and individual level. At the national level, it shows the lack of a unified framework for reporting COVID-19 data in India, and highlights the need for a central agency to monitor or audit the quality of data reporting done by the states. Without a unified framework, it is difficult to aggregate the data from different states, gain insights, and coordinate an effective nationwide response to the pandemic. Moreover, it reflects the inadequacy in coordination or sharing of resources among the states. The disparate reporting score also reflects inequality in individual access to public health information and privacy protection based on the state of residence.

**Supplementary Information:**

The online version contains supplementary material available at (10.1186/s12889-021-11054-7).

## Background

India reported its first case of COVID-19 in the state of Kerala on January 30, 2020. Since then the disease has been reported in several other states and union territories (UTs) of India. As of July 18, 2020, the Ministry of Health and Family Welfare (MoHFW) of India reported over a million COVID-19 confirmed cases and over twenty-six thousand COVID-19 deaths in the country [1]. India is a developing nation and has the second largest population in the world. India is also a democracy with 28 states and 8 union territories. Therefore, coordinating an effective response to the pandemic, across all the regions, presents a unique and unprecedented challenge to India.

Both the central and state governments in India have introduced multiple measures and interventions for the containment of COVID-19 [2]. It is well known that public adherence to these measures and interventions is essential for managing the pandemic [3]. In order to keep the public informed about the ongoing situation, the states in India have been reporting COVID-19 data collected through surveillance programmes. As per World Health Organization’s (WHO) guidance, surveillance is essential to monitor trends in COVID-19, to conduct risk assessments, and to guide preparedness and response measures [4]. Reporting relevant data in a timely, transparent, and accessible manner is crucial during a pandemic [3]. The advantages of such a timely reporting are atleast two-fold. First, it fosters trust between the government and the public and, thereby ensures public cooperation. Second, it enables the scientific community to rapidly and continually study the reported data to gain insights and propose better containment measures and policies. A schematic of a good data reporting system that we envision is shown in Supplementary section S1 of Additional file 1.

**Fig. 1:**
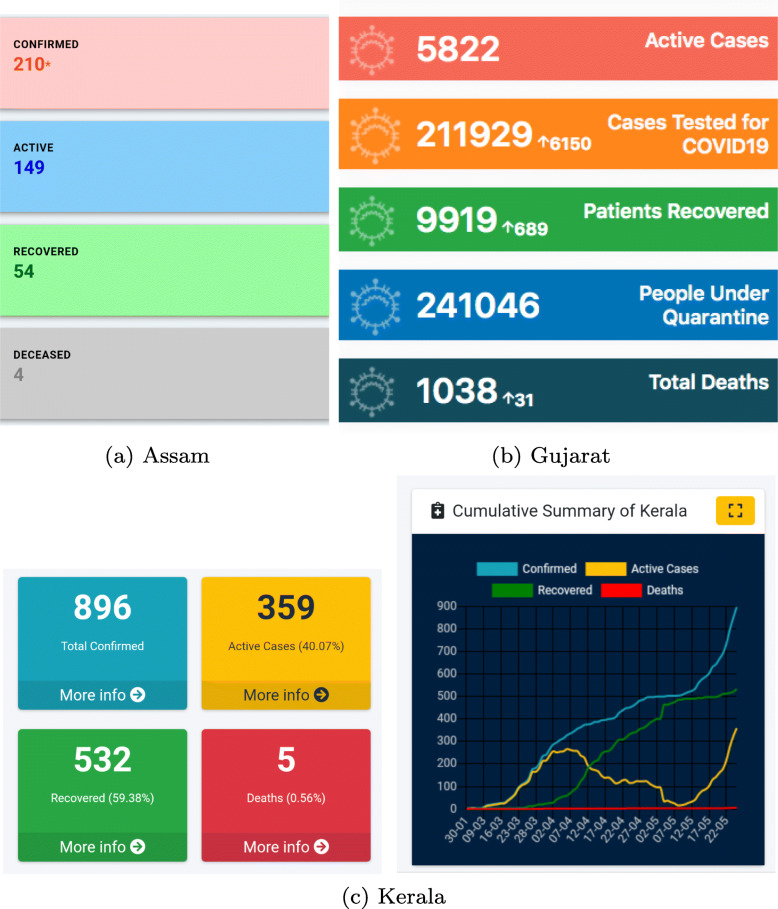
Screenshots from the government COVID-19 web pages of Assam, Gujarat, and Kerala displaying cumulative data. Kerala is the only state among these three states to provide both a textual summary and trend graphics

Each Indian state^1^ has its own mechanism (daily bulletins, dashboards, etc.) for reporting COVID-19 surveillance data. The content and format of the data reported through these bulletins/dashboards vary substantially from state to state [5, 6]. Figure 1 shows how total (cumulative) numbers are reported by three different states in India. Notice how Assam and Gujarat report just the total numbers, whereas Kerala reports the numbers and their trend graphics. In addition to reporting the numbers, providing trend graphics is essential because it concisely represents the data, and makes it more interpretable and accessible to the general public. In the rest of this section, we give a brief overview of data reporting quality, data quality, data visualization, and a crowdsource initiative for reporting COVID-19 data. We conclude the section with a summary of our objectives and contributions.

### Quality of data reporting

A leading Indian English newspaper, The Hindu, published an article showing variance in COVID-19 data reporting across the states in India [7]. However, their analysis has atleast three main limitations. First, it provides only a high-level summary of the variance in data reporting and is limited to 21 states. Second, the article focuses only on data reported in the health bulletins. Third, they don’t provide a quantitative analysis. Janiaud and Goodman developed less granular metrics to assess reporting quality in the U.S. states [8].

### Data quality

Data quality is a multidimensional concept with dimensions such as, accuracy, accessibility, completeness, interpretability, relevancy, and timeliness [9]. There are frameworks for data quality assessment that are motivated by what data quality means to the consumers of data [10, 11]. Although there is an overlap between quality of data and quality of data reporting, they are not quite the same. Accuracy is a crucial aspect in data quality. However, while measuring the quality of data reporting, the emphasis is not on the accuracy of data, instead it is on the presence or absence of a piece of information and the format in which it is reported.

### Data visualization

Visualization is critical for understanding data. Excellent statistical graphics communicate complex ideas with clarity, precision, and efficiency [12]. The best practices in creating statistical graphics are discussed extensively in the books by Cleveland and Tufte [12, 13]. There is also rich literature on developing effective real-world dashboards [14], and interactive visualization for the web [15].

### Crowdsource initiative for COVID-19 data reporting

covid19india.org is a volunteer driven crowd-sourced tracker for COVID-19 cases in India. They collect and curate COVID-19 data from all across India, from a variety of sources, including but not limited to state government websites [16, 17]. The curated data is reported on their website in the form of tables, trend graphics, and color-filled maps. The covid19india team has an active page on Twitter with more than 100 thousand followers. Based on the number of followers and the kind of questions they ask (see Supplementary section S3, Additional file 1), it is evident that people are seeking granular COVID-19 data on a daily basis. This crowdsource initiative is a commendable example for public participation during a crisis. Nevertheless, it is not sufficient, and does not replace the need for clear and consistent government official reporting for the following reasons. The initiative is volunteer driven and hence accountability is not guaranteed in the event of an error or lapse in reporting. Moreover, their sources for data include social media, which are noisy.

### Objectives and contributions

The variance in reporting COVID-19 data across the Indian states raises two key questions. First, what is the minimal data that the public needs to know to understand the gravity of the situation and cooperate with the government? Second, how different is the quality of data reporting from one state to another? In this paper, we answer the two aforementioned questions by developing a systematic framework to evaluate the quality of COVID-19 data reporting. We then use it to assess the quality of reporting done by the states in India. We compare the quality of reporting of each state to its Sustainable Development Goal India Index for Good Health and Well-Being (SDG3-II), reported by NITI Aayog [18, 19]. Based on our framework we also provide a minimal template that the states can use for daily COVID-19 data reporting (given in Supplementary section S2, Additional file 1). We also present our findings on an interactive Tableau dashboard that’s easily accessible [20].

## Methods

We developed a set of metrics to score the quality of “COVID-19 data reporting” done by the states in India. These metrics are shown in column 2 of Table 1. The metrics are further grouped into four categories: availability, accessibility, granularity, and privacy, as shown in column 1 of Table 1. Using these metrics, we examine the quality of reporting for five items relevant to COVID-19. They are confirmed, deaths, recovered, quarantine and intensive care unit (ICU) cases. These are called as report items and appear as column headers in the scoring table. The report items and the metrics for availability and granularity are based on WHO’s recommendations to the different nations for reporting surveillance data to them [4]. The choice of accessibility metrics reflect our belief that the format in which pandemic data is reported/presented should ensure that the key public health messages reach a wider audience beyond the scientific community. For example, the widely used phrase “flatten the curve” is easily understood if data is presented in the form of trend graphics.

**Table 1:** CDRS Scoring metric table

		Report Item				
Category	Metric	Confirmed	Deaths	Recovered	Quarantine	ICU
Availability	Total	{0,1}	{0,1}	{0,1}	{0,1}	{0,1}
	Daily	{0,1}	{0,1}	{0,1}	{0,1}	{0,1}
	Historical data	{0,1}	{0,1}	{0,1}	{0,1}	{0,1}
Accessibility	Ease of access	{0,1}				
	Availability in English	{0,1}				
	Trend graphics – Total	{0,1}	{0,1}	{0,1}	{0,1}	{0,1}
	Trend graphics – Daily	{0,1}	{0,1}	{0,1}	{0,1}	{0,1}
Granularity	Stratified by age	{0,1}	{0,1}	{0,1}	**–**	{0,1}
	Stratified by gender	{0,1}	{0,1}	{0,1}	**–**	{0,1}
	Stratified by comorbidities	{0,1}	{0,1,2}	{0,1}	–	{0,1}
	Stratified by districts	{0,1}	{0,1}	{0,1}	{0,1}	{0,1}
Privacy	Compromise in privacy	{-1,1}				

This table is filled for each state by inspecting the COVID-19 data reported by that state. The entry within a cell in the table lists all the possible values with which that cell can be filled. Broadly, a 0 represents an unreported item, and a 1 represents a reported item

The choice of report items and metrics (in particular privacy) are also influenced by the questions posed in the paper, “Transparency during public health emergencies: from rhetoric to reality” [3]. The paper identifies three YES/NO questions to help in deciding whether or not to release a piece of information related to a public health emergency. These questions seek to understand the role of a piece of information in: (i) reducing the spread of a disease, (ii) emergency management decision making process, and (iii) compromising privacy or stigmatization of specific groups of people or both.

The report items also represent five possible stages through which an individual can go through during a pandemic. For example, an individual could move from the stage of being under quarantine, to being a confirmed case, and from there could recover in a couple of weeks, or if the situation worsens, could move to ICU. At the time of this study, all confirmed COVID patients in India were hospitalized and treated in one of the following facilities: COVID Care Centers, Dedicated COVID Health Centers or Dedicated COVID Hospitals [21].

Each “Metric - Report item” pair is a data reporting quality indicator (variable). Overall there are 45 indicators in our framework across the four scoring categories. It is important to note that neither the list of metrics, nor the list of report items used in our scoring table are exhaustive. It is a representative minimal set. We define the report items as follows.

1. Laboratory Confirmed: refers to individuals who tested positive for COVID-19.
2. Deaths: refers to individuals who passed away while being COVID-19 positive [22, 23].
3. Recovered: refers to individuals who recovered from COVID-19.
4. Quarantine: refers to individuals who are under quarantine either at home or specific government facilities. The definition of who should be quarantined and for how long has evolved during the course of pandemic in India.
5. ICU: refers to COVID-19 positive individuals who are under treatment in an ICU.

In our framework, we do not make any distinction between methods used to define a case as confirmed (RT-PCR, rapid antigen test, etc.) or recovered (by symptoms or lab test). The methods used have changed over time and across states in India. However, to the best of our knowledge, at the time of this study RT-PCR test was used through-out India to determine a case as confirmed or recovered [24].

### Scoring categories

In this section we give an overview of the four scoring categories.

1. **Availability of data**. During a pandemic, few generic questions that people seek to answer are: “How are we doing?”, “How do we know how we are doing?”, “How long will this last?”, “How do the numbers from today compare with yesterday’s?”, “How many people have tested positive so far”, and so on. With such questions in mind we measure the availability of data by checking if the total, daily, and historical data are available for each report item.
2. **Accessibility of data**. Data should not only be available, it should also be easily accessible. We measure the accessibility of data based on ease of access, availability in English, and the presence of trend graphics. Ease of access refers to the ease of getting to the web page where data is reported. Research has shown that trend graphics are superior than tables for identifying and displaying trends [25]. A good visual concisely represents the data and makes it more interpretable and accessible to the general public. Therefore, to measure accessibility we also check, if a trend graphic of total and daily are available for each report item. However, we do not assess the attributes of a graphic such as shape (length to height ratio), line weight, choice of colors, font size of text, and whether the graphic is interactive or not.
3. **Granularity of data**. Granularity refers to the stratification of the total number for each report item. We check if the total is stratified by age, gender, comorbidities, and districts. Recent studies have shown the role of age, gender, and comorbidities in influencing the outcome of a COVID-19 positive individual [26–28]. As per the Indian Council of Medical Research (ICMR) specimen referral form for COVID-19, data on age, gender, district, and pre-existing medical conditions are collected for each person being tested [29]. Therefore, aggregating and then stratifying that information should be straightforward. At a higher level stratified information is useful in the following ways. (i) District level data keeps the public informed about the gravity of situation in their neighborhood. (ii) People can self-identify how susceptible they are to get infected and hence take the necessary precautions. For example, granular data can answer questions of the kind, “I’m 65 and healthy, should I be worried?” (iii) Scientific community can study the effect of factors like age, gender, and comorbidities on contracting the disease, its progression, and the outcome.
4. **Privacy of data**. Data released by the government should include only the minimum information necessary to conduct public health activities [30]. It should not contain any personally identifiable information. Violating privacy by releasing personally identifiable information can have the following consequences. (i) It can discourage people from cooperating with the government, thereby hurting public health rather than helping. (ii) Women can be victims of harassment calls when their phone number is released. A study by Truecaller shows that, in general, 8 out of 10 women in India receive harassment and nuisance calls [31]. Releasing phone numbers can further amplify the general trend. (iii) Discrimination and stigmatization of specific groups of people [32–34].

### Scoring data curation

We evaluated the quality of “COVID-19 data reporting” done by the states during the two week period from May 19 to June 1, 2020 by recording information in Table 1. Hereafter, this recorded data is referred to as the *scoring data* and the two week period is referred to as the *scoring period*. By May 18, India was already under lockdown for more than 50 days. This is sufficient time for state governments to develop a good data reporting system. The fact that India had reported 96 thousand confirmed cases by then made it all the more important to warrant a high quality data reporting system. Therefore, our choice of scoring period is reasonable and the scoring data curated during this period captures a quasi-steady state for reporting. States that reported less than 10 total confirmed cases as of May 18, were excluded from the study. The excluded states were: Arunachal Pradesh, Dadra and Nagar Haveli and Daman and Diu, Lakshadweep, Manipur, Mizoram, Nagaland and Sikkim. After the exclusion we were left with 29 states for assessment. In each of these states, as per Wikipedia, the first case was reported atleast 30 days prior to May 19.

The authors applied the scoring criteria in Table 1 to each state and reached a consensus on the curated scoring data. For each state, the authors checked the government and health department websites for COVID-19 data to fill the scoring table. If no data was available on either of those websites then a google search was done to find other official sources. During the process if any official website was found to contain COVID-19 data, then that was used to fill the scoring table. Social media websites like Twitter and Facebook were excluded for the following reasons. First, there are multiple social media platforms. Expecting people to be on the right platform and following the right person to obtain relevant public health information is unreasonable. Second, relevant information can easily get lost amid several posts. Third, obtaining historical data by scrolling through the feed is practically impossible.

We begin the scoring data curation with a score of 0 for each indicator. We then check the COVID-19 data reported by the state and fill the scoring table as follows. Indicators corresponding to total, daily and historical metrics are assigned a score of 1 if total, daily and historical data are available. Indicators for trend graphics are assigned a score of 1 if the corresponding trend graphic is present. If all the data is available in English then the corresponding indicator gets a score of 1. The ease of access indicator is scored 1 if the web page where data is reported is linked from either the state government website or the state health department website. Indicators representing stratification by age, gender, comorbidities, and districts are given a score of 1 if the reported data contains total for a report item disaggregated by these variables. For deaths, the comorbidity indicator is assigned an additional score of 1 if patient specific comorbidities are reported. Finally, the privacy indicator is given a score of -1 if the data reporting compromises privacy by releasing personally identifiable information such as name, address or phone number. If privacy is not compromised a score of 1 is assigned. Indicators that are not applicable for a state are marked as ‘NA’. For example, stratified by districts is not applicable to Chandigarh, as it doesn’t have any districts. For more details on the scoring metrics and scoring data curation refer to Supplementary sections S4 and S5 of Additional file 1.

### Score calculation

For each state we calculate four categorical scores — availability, accessibility, granularity, and privacy, and an overall score, which is referred to as the COVID-19 Data Reporting Score (CDRS). Categorical scores for a state are calculated by summing the entries corresponding to that category in the scoring table. The normalized score *N* in category *c* for state *s*, is then calculated as,

$$N(c, s) = \frac{T(c, s)}{M(c,s) - m(c,s)}, $$

where *T* is the total score and, *M* and *m* are the maximum and minimum possible scores. CDRS is the normalized sum of these four categorical scores and is given by,

$$\text{CDRS}(s) = \frac{\sum_{c\in C}T(c,s)}{\sum_{c\in C} M(c,s)} $$

where *C* denotes the set of all categories. CDRS ranges from 0 (lowest quality) to 1 (highest quality). For numerical examples of CDRS and categorical score calculation, see Supplementary section S7, Additional file 1.

**Fig. 2:**
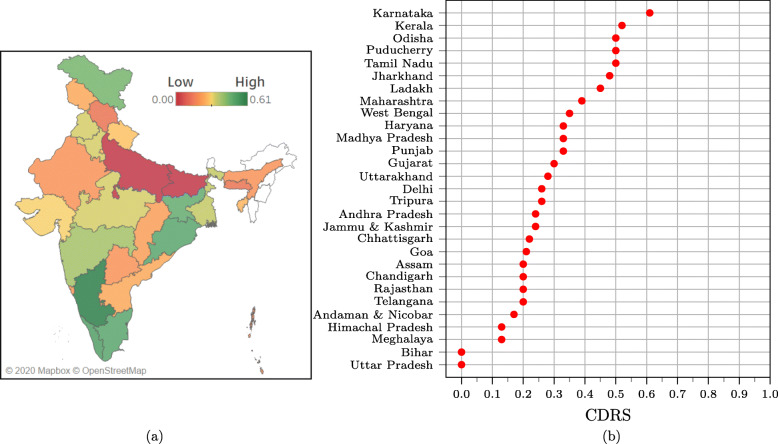
(a) Map showing CDRS across the states and UTs in India. States and UTs that were excluded from our study are filled with a white color. The map clearly shows the geographical disparity in COVID-19 data reporting in India. The map was generated using Tableau Desktop software version 2020.2.1 and the boundary information for regions in India was obtained as shapefiles from Datameet (http://projects.datameet.org/maps/). (b) A dot plot showing the spread of CDRS values. On the *y*-axis, states are sorted in the decreasing order of CDRS

CDRS and the normalized categorical scores for the states are available in Supplementary Table S3 of Additional file 1. The normalized scores for availability, accessibility, and granularity range from 0 (lowest value) to 1 (highest value). The normalized privacy score is 0.5 when there is no violation of privacy and −0.5 otherwise. Privacy score is not applicable for states that do not report any data. For all the score calculations, normalization was adjusted to account for not applicable (‘NA’) entries in the scoring data (see Supplementary section S5, Additional file 1). We also present CDRS as a color map as shown in Fig. 2. The map was generated using Tableau Desktop software version 2020.2.1 and the boundary information for regions in India was obtained as shapefiles from Datameet [35].

### CDRS and SDG3-II

SDGs are a set of 17 global goals to achieve by 2030, set by the United Nations in 2015 [18]. The SDG India Index 2019–2020, developed by NITI Aayog, is a framework to measure the progress of states based on their performance across SDGs [19]. The framework was developed using 100 indicators across 54 SDG targets. SDG3-II measures the performance of states on the third SDG, which is, “Good Health and Well-Being for all”. The value for SDG3-II ranges from 0–100, where 100 implies that the state has achieved the target set for the year 2030. The indicators used by NITI Aayog in their framework to calculate SDG3-II are listed in Supplementary section S6 of Additional file 1. We assess the Pearson and Spearman’s rank correlation between CDRS and SDG3-II using the corr function in Matlab R2019a.

## Results

A COVID-19 Data Reporting Score (CDRS), and four normalized categorical scores were calculated for 29 states in India. In each of these states, the first case was reported atleast 30 days prior to our assessment. Thus, they had atleast a month’s time to build a high-quality data reporting system. Our results and conclusions should be viewed and interpreted in light of this time frame.

There is a strong disparity in the quality of COVID-19 data reporting done by the different states. The five number summary of CDRS is, min = 0.0, first quartile = 0.2, median = 0.26, third quartile = 0.41, and maximum = 0.61. The disparity can be clearly seen in Fig. 2, which shows the CDRS for the different states, both as a color-filled map and as a dot plot. Visuals for the normalized availability, accessibility, granularity, and privacy scores are available in Supplementary Figure S3, Additional file 1.

The best data reporting is done by Karnataka (0.61), Kerala (0.52), Odisha (0.50), Puducherry (0.50), and Tamil Nadu (0.50). All these states provide a dashboard that shows the trend of COVID-19 data graphically. They also provide district wise stratification of the total confirmed, recovered, and deaths due to COVID-19. However, not all of them stratify the data according to age, gender, and comorbidities, the factors that are known to have a correlation with the COVID-19 fatality rate [26–28]. Even Karnataka, the state with highest CDRS, has further scope for improvement.

**Fig. 3:**
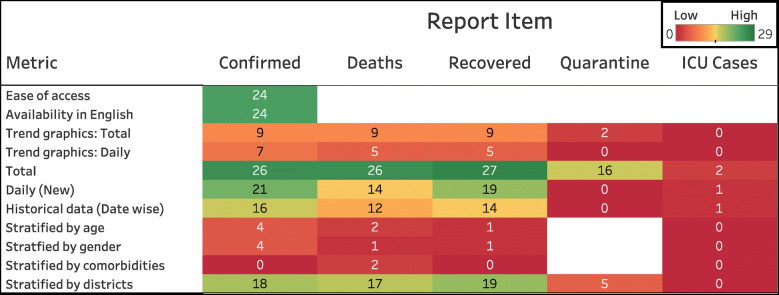
Table shows the number of states reporting an item out of twenty-nine states/UTs. Twenty-six of these report the total number of COVID-19 confirmed cases and deaths, and twenty-seven states report the number of recovered individuals. Only a handful of the states publish cumulative data stratified by age, gender, and comorbidities

On the other hand, Uttar Pradesh (0.0), Bihar (0.0), Meghalaya (0.13), Himachal Pradesh (0.13), and Andaman and Nicobar Islands (0.17) rank at the bottom. Uttar Pradesh and Bihar do not publish any COVID-19 data on their government or health department website. However, Bihar seems to release some data on Twitter. See Supplementary section S9 of Additional file 1 for more details. Himachal Pradesh, Meghalaya, and Andaman & Nicobar Islands, report just the total count for few report items. Daily count, trend graphics and granular data are not reported by these states. For details on the number of states that report a specific information, refer to Fig. 3.

**Fig. 4:**
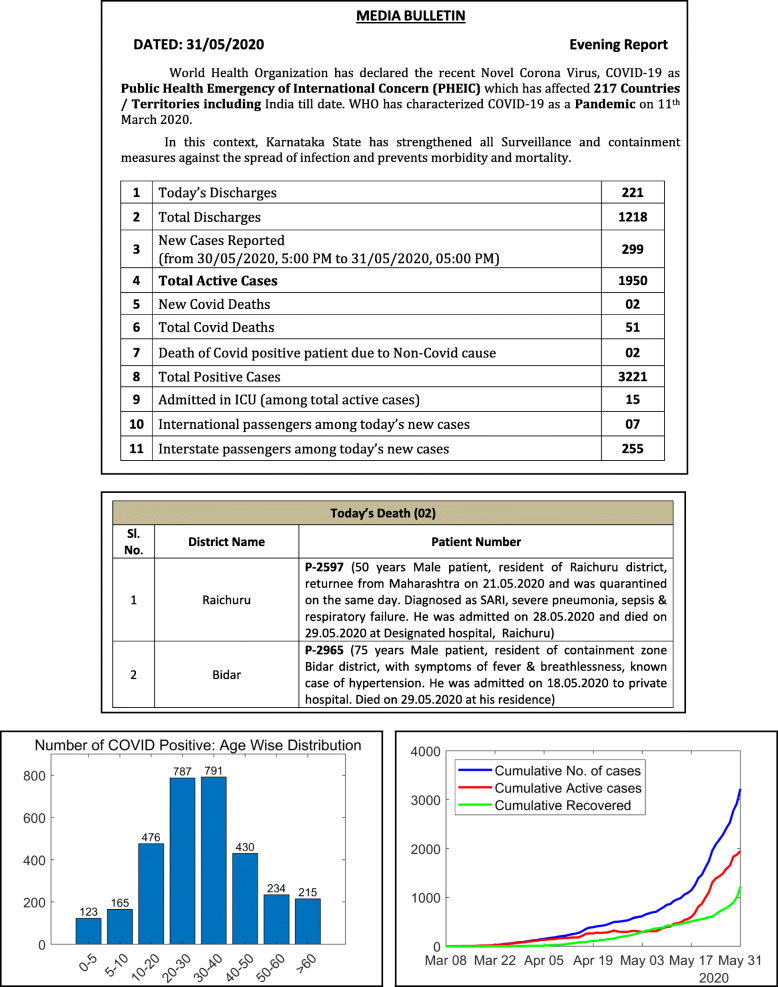
Bulletin and visualization provided by the Karnataka government on May 31, 2020 as examples of high-quality COVID-19 data reporting

Figure 4 shows the bulletin and visualization provided by Karnataka on May 31, 2020. Karnataka’s COVID-19 page is linked from the state government’s website. The state releases a health bulletin and a state war room bulletin everyday, and also maintains a dashboard. The bulletins are available in English, and provides information on the total confirmed, deaths, recovered, quarantined, and active ICU cases. The bulletins also report some daily (new) data, and some data stratified by age, gender, and districts. In addition, the demographics and comorbidity data are reported for each deceased person. Trend graphics are available either through the bulletins/dashboard.

Karnataka and Punjab score the highest in availability. Both these states report the daily and total numbers for confirmed, deceased and recovered cases. They also report COVID-19 cases in Intensive Care Units (ICUs). Historical data is available for both the states in the form of daily bulletins. Among the states that report data, Assam, Himachal Pradesh, and Meghalaya score the lowest for availability. This is because they report only the total count for confirmed, deceased, and recovered. A screenshot of the data reported by Assam is shown in Fig. 1a.

COVID-19 data can be accessed from the state’s official websites for 83% of the states evaluated. Only 10 states make the data more accessible by providing a visual representation of the trend. Karnataka and Kerala score the highest (0.75) in accessibility. These states provide trend graphics for both total and daily data, for the confirmed, deceased, and recovered cases. Figure 1c shows the screenshot of a trend graphic displayed on Kerala government’s COVID-19 dashboard.

In general, the worst categorical scores are for granularity. Even Jharkhand, the top state in this category, scored only a 0.50, while the median normalized granularity score is 0.17. For more details on the granular data published by Jharkhand refer to Supplementary section S8 of Additional file 1. Karnataka and Tamil Nadu are the only states to provide details of death (including comorbidity information) for each deceased person. The following states do not report any data stratified by age, gender, comorbidities, or districts: Andaman and Nicobar Islands, Andhra Pradesh, Bihar, Chandigarh, Delhi, Goa, Himachal Pradesh, Meghalaya, Telangana and Uttar Pradesh.

**Fig. 5:**
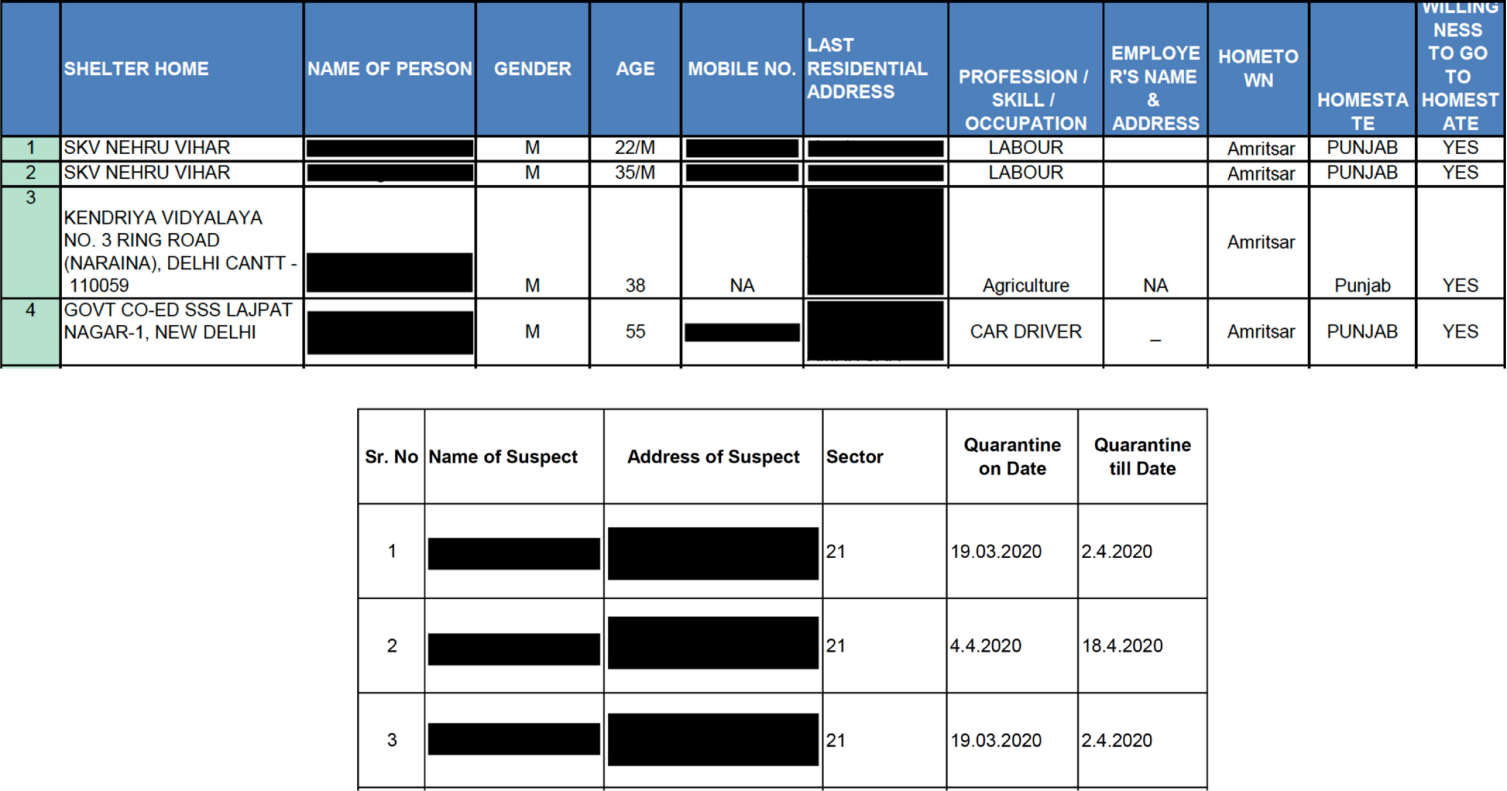
Screenshots of the documents published by Punjab (top panel) and Chandigarh (bottom panel) that contain individually identifiable information of people under quarantine. The Names and addresses of individuals were reported on the official website and we blacked it out here

Among the states that were assessed 27 reported some data. Privacy doesn’t apply to states that do not report any data. Among the 27 states that report some data, all of them except Chandigarh and Punjab, report de-identified information and do not violate the privacy of the people residing in their state. Chandigarh has released name and residential address of people under home quarantine. Punjab has released name, gender, age, and mobile number of persons inbound to the state from New Delhi on May 10, 2020. Figure 5 shows screenshots from the documents published in the government websites of Punjab and Chandigarh that contain personally identifiable information.

According to MoHFW as of May 18, 2020, the total number of confirmed cases in India were about ninety-six thousand. The top ten states when sorted according to the number of confirmed cases contributed to a staggering 91% of the total confirmed. These ten states are shown in Figure S5 (see Supplementary, Additional file 1) above the horizontal dashed line. Tamil Nadu is the only state among those ten states with a CDRS in the 75th percentile. Figure S2 of the Supplementary (in Additional file 1) shows a scatter plot that displays the relationship between CDRS and SDG3-II. A positive correlation was observed between CDRS and SDG3-II (Pearson correlation: *r*=0.630,*p*=0.0003; and Spearman’s rank correlation: *r*=0.578,*p*=0.001).

## Discussion

The scoring data curated in this study identifies what data each state in India is reporting and its format. We observed that the majority of states are not reporting the number of confirmed cases and deaths stratified by age and gender. Hoffmann and Wolf make similar observations in the data reported from European countries, and call for standardized data collection by national health authorities [36]. They report that as of July 6, 2020, data on ages were incomplete for France, and completely missing for Armenia, Luxembourg, North Macedonia, Turkey, Serbia and Bosnia-Herzegovina. As of May 1, 2020, even the US lacked a shared standard for COVID-19 data reporting, resulting in a large variation in the quality of reporting across the states and counties [37].

The disparity in CDRS across the states highlights three important findings at the national, state, and individual level, respectively. First, it shows the lack of a unified framework for reporting COVID-19 data in India, and the need for a central agency to monitor or audit the quality of data reporting done by the states [5]. Without a unified framework, it is difficult to aggregate the data from different states, gain insights from them, and coordinate an effective nationwide response to the pandemic. Not just that, unified high-quality data reporting also signifies transparency and hence increases public trust in the government. Containment becomes easier when the public is well-informed.

Second, it reflects the inadequacy in coordination or sharing of resources among the states in India. Coordination among states is particularly important as more people start moving across the states in the coming months. While it might not be possible for all the states to setup a high-quality dashboard in a short time, states can nevertheless seek help and learn from the best data reporting practices followed by the other states.

Third, the disparate reporting score also reflects inequality in individual access to public health information and privacy protection based on the state of residence. The inequality highlights that the state-level efforts do not align with the central government’s vision of treating public health data as a public good, within the legal framework of data privacy, as described in the 2018–19 economic survey of India [38]. We cannot stress enough about the importance of respecting the privacy of all citizens. One might argue that providing residential address of people under home quarantine is helpful to identify areas to avoid in a locality. However, the same information can be conveyed using hotspot maps that can be generated using geomasking techniques to protect privacy [39].

The observed positive correlation between CDRS and SDG3-II suggests that governments which are making more progress toward the “sustainable development goal of good health and well-being” also tend to have better COVID-19 data reporting. The scatter plot of CDRS versus total confirmed COVID-19 cases shown in Figure S5 (see Supplementary, Additional file 1) suggests that states with the highest number of cases also tend to have poor COVID-19 data reporting, which could further exacerbate the pandemic challenges.

## Conclusions

Overall, our scoring framework and CDRS together helps in identifying the differences in the quality of COVID-19 data reporting across India. In addition to revealing the disparity in the quality of reporting, CDRS also highlights that there is tremendous scope for all states to improve. The categorical scores enable states to identify their strengths and weaknesses. In each category, states can learn from their peers and improve their quality of reporting. States that score high in a category can serve as role models to the other states.

Although we focus on India in this paper, the scope of the scoring framework is not limited just to India. It can be adapted to other countries. Within India, our scoring framework could also be applied at the district level to evaluate the quality of data reporting across districts within a state. A future work is to conduct the same study a few months later and assess the change in the quality of data reporting.

## Limitations

Some of the limitations of our study are as follows. (i) We did not include the reporting of testing data in our framework. This is because the degree of relevance of testing data in understanding the course of pandemic depends on whether testing was done on a scientific random sampling basis or not. (ii) Some states in India have developed mobile applications for COVID-19. We were unable to download and install them due to geographical restrictions. Therefore, our study doesn’t consider data that states might be reporting through these mobile applications. (iii) To calculate the scores, we assign an equal weight to each reported item. One could potentially assign unequal weights, however, finding an appropriate set of unequal weights is beyond the scope of this work.

## Supplementary Information

**Additional file 1** Schematic of a good data reporting system (section S1); Template for daily COVID-19 data reporting (section S2); Twitter page of covid19india.org (section S3); Details of the scoring metrics (section S4); Scoring process (section S5); SDG3-II (section S6); Categorical scores (section S7); Screenshot from a bulletin published by Jharkhand (section S8); Additional notes on a few states (section S9); Sources for scoring data (section S10); Total confirmed COVID-19 cases as of May 18, 2020 (section S11).

## Data Availability

The curated scoring data is publicly available at https://github.com/varun-vasudevan/CDRS-India. The data was curated from publicly available sources listed in Supplementary section S10 of Additional file 1.

## Abbreviations

COVID-19: Coronavirus disease 2019
UT: Union Territory
MoHFW: Ministry of Health and Family Welfare
ICMR: Indian Council of Medical Research
ICU: Intensive Care Unit
WHO: World Health Organization
CDRS: COVID-19 data reporting score
SDG: Sustainable Development Goals
SDG3-II: Sustainable Development Goal 3 - India Index

## Footnotes

1 From here on, unless specified otherwise, the word state refers to both a state and union territory in India.
