# Supplementary material for: Disparity in the quality of COVID-19 data reporting across India
Source: BMC Public Health. 2021 Jun 24;21:1211. doi: 10.1186/s12889-021-11054-7 (PMC8223181; doi:10.1186/s12889-021-11054-7)
Supplement: Supplementary file 1 — Additional file 1 Schematic of a good data reporting system (section S1); Template for daily COVID-19 data reporting (section S2); Twitter page of covid19india.org (section S3); Details of the scoring metrics (section S4); Scoring process (section S5); SDG3-II (section S6); Categorical scores (section S7); Screenshot from a bulletin published by Jharkhand (section S8); Additional notes on a few states (section S9); Sources for scoring data (section S10); Total confirmed COVID-19 cases as of May 18, 2020 (section S11). [file 12889_2021_11054_MOESM1_ESM.pdf]

Additional File 1

Supplementary Materials for
Disparity in the quality of COVID-19 data reporting across India

Varun Vasudevan*, Abeynaya Gnanasekaran, Varsha Sankar, Siddarth A. Vasudevan, James Zou
*Corresponding author. Email: devan@stanford.edu

S1 Schematic of a good data reporting system

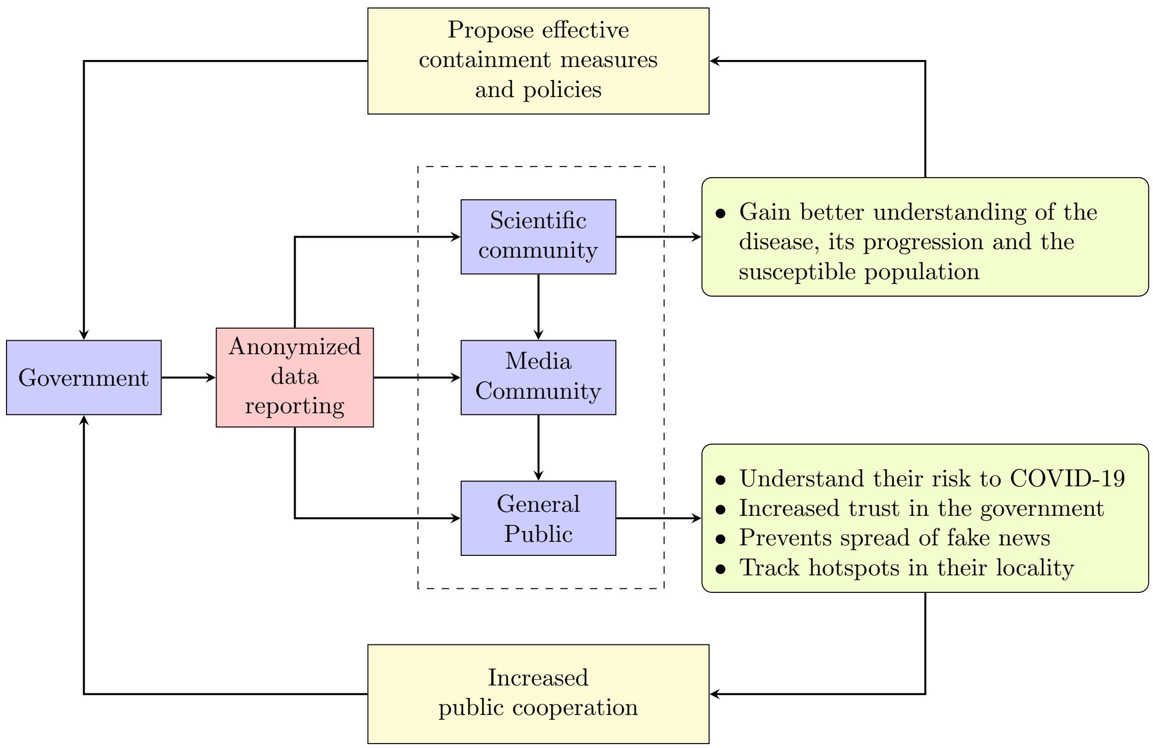

Figure S1: A schematic summarizing the positive outcomes of a good data reporting system that we envision.

Figure S1 shows the schematic of a good data reporting system that we envision. As seen from the schematic, data reported by the government is consumed by the general public and the scientific community. The general public consumes data either directly from the government sources or through news media. As shown in Figure S1, high quality data reporting by the government creates a positive feedback loop that in turn helps the government contain the pandemic better.

S2 Template for daily COVID-19 data reporting

**State Name**	
**Date**	

	**New cases**	**Total cases**
**Confirmed**		
**Deaths**		
**Recovered**		
**Quarantine**		
**Active ICU cases**		

**Age stratification**				
**Age brackets**	**Total confirmed**	**Total deaths**	**Total recovered**	**Active ICU cases**
<5				
5-14				
15-24				
25-34				
35-44				
45-54				
55-64				
65-74				
75-84				
85+				

**Gender stratification**				
**Gender**	**Total confirmed**	**Total deaths**	**Total recovered**	**Active ICU cases**
Male				
Female				
Other				
Unknown				

**District stratification**					
**Districts**	**Total confirmed**	**Total deaths**	**Total recovered**	**Total quarantine**	**Active ICU cases**
District 1					
District 2					
District 3					
District 4					

**Comorbidity stratification**				
	**Total confirmed**	**Total deaths**	**Total recovered**	**Active ICU cases**
1 or more comorbidities				
None				
Unknown				

**Age, gender, comorbidity details of new deaths**				
	**Age**	**Gender**	**District**	**List of comorbidities**
P1				
P2				
P3				

S3 Twitter page of covid19india.org

The following are a set of sample questions[1] asked on the twitter page of covidindia19.org.

(a) Request for data at the district level.
- https://twitter.com/craomumbai/status/1261156322860388352
- https://twitter.com/HarshuSamnani/status/1246725069646098434

(b) Request for death data stratified by age, gender, comorbidities, and districts.
- https://twitter.com/ramesh_basil/status/1248673419920461825
- https://twitter.com/nikhilvaishy65/status/1264051034688651265
- https://twitter.com/UPisute/status/1259667770427359232

S4 Details of the scoring metrics

In this section we provide additional information on each scoring metric listed in Table 1 in the main manuscript to elucidate what a metric is checking/assessing and its importance in the context of data reporting.

- Total: Total refers to the total as of a given date. This metric checks the availability of total information for each report item. Presence of the total information is assigned a score of 1 and absence a 0 as shown in Table 1 in the main manuscript. Cumulative data is useful in multiple ways. For example, using total confirmed one can calculate the percentage of population that has been infected and the doubling time of the disease.
- Daily (New): This metric checks the availability of new numbers for each report item on a daily basis. A score of 1 is given for a report item if the new number is available and 0 otherwise.
- Historical data: This refers to the availability of the historical daily data for each report item. Availability of historic data is crucial in determining whether or not the epidemic curve is flattening. A score of 1 is given if historical daily data is available for a report item and 0 otherwise.
- Ease of access: The data is categorized as easily accessible (represented by a 1) if the web page where data is reported is linked from either the state government website or the state health department website.
- Availability in English: Data is marked as available in English (represented by a 1), only if all the items marked as reported in the scoring table are available in English. When the data is reported in English, it is available to a wider audience who speak different regional languages.
- Trend Graphics – Total: This refers to the time-series line chart of the total of a reported item. Date is represented on the horizontal x-axis and total value on the vertical y-axis. The height and slope of the line allows us to see the trends. A score of 1 is given if the trend graphic is present and 0 otherwise.
- Trend Graphics – Daily: This is the same as trend graphics for total, but with daily (new) numbers on the y-axis.
- Stratified by age: This checks if the total for a report item is split into age brackets. For example, a state could stratify the total number of deaths and report the number of deaths

[1] These tweets were last accessed on June 28, 2020.

in the age groups <5, 5–14, 15–24, . . ., 75–84 and 85+. This information is helpful to identify and protect the vulnerable population. A score of 1 is given if data stratified by age is available and 0 otherwise.

- Stratified by gender: It refers to the stratification of the total for a report item by gender. Current global data shows that the infection and mortality rate are high among men.[1] Such inferences would not have been possible if gender stratification of the infected population was not reported. Furthermore, monitoring local trends in the data is useful to improve our understanding of the infection by either confirming or contradicting the global data. A score of 1 is given if data stratified by gender is available and 0 otherwise.
- Stratified by comorbidities: It refers to the stratification of the total for a report item by comorbidity. To keep it simple, if binary stratification (presence/absence of comorbidity) is reported we record a 1 in the scoring metric table. For the case of deaths, a score of 1 is recorded if either of the following information is reported: (i) binary stratification (ii) patient specific comorbidities for each death. If both are reported, a score of 2 is assigned.
- Stratified by districts: It refers to the stratification of the total for a report item by districts within the state. For the general public, stratification by districts is way more important than stratification by age, gender and comorbidity. District level information is helpful for the public to understand the effect of the pandemic in their neighborhood, and to cooperate and adhere with government policies and interventions. District level information is also useful for manufacturers of healthcare equipment like personal protective equipment and ventilators, to decide on resource allocation and supply chain logistics. A score of 1 is given if data stratified by districts is available and 0 otherwise.
- Compromise in privacy: This metric checks if any personally identifiable information related to individuals who are quarantined or tested positive for COVID-19 are published online by the government. Examples of identifiable information include name, address, and mobile number. As explained in the scoring categories section of the main paper, releasing personally identifiable information can have dire consequences. A score of $+1$ or -1 is entered in the scoring table to indicate "no violation" or "violation" of privacy respectively. We give a negative score for privacy violations because it is detrimental.

S5 Scoring process

During the scoring period, data was curated for each state by filling the scoring metric table shown in Table 1 in the main manuscript by following the scoring metrics described in section S4. The steps followed to fill the scoring metric table for each state are as follows.

- Authors VV and AG jointly checked the government and health department websites of the state for COVID-19 data on an arbitrary day during the scoring period. Data available on these websites was used to fill the scoring table. If no data was available on either of those websites then a google search[2] was done to find other official sources. During the process if any official website was found to contain COVID-19 data, then that was used to fill the scoring table. Social media websites like Twitter and Facebook were excluded. The links to official government websites reporting data are available in section S10.
- Two other authors VS and SAV independently verified the entries in the scoring table based on the data reported by the state on another date during the same time period, by following the procedure described above. Any discrepancy/mismatch was noted down for further review by authors VV and AG. The set of states verified by VS and SAV were mutually exclusive.
- For the states that reported data in a regional language that none of the authors could read, external help from a native speaker of that language was obtained to fill the scoring table. There were two states in this category.
- After the scoring period authors VV and AG did a final pass over all the states. During the final pass VV and AG did the following.
  - Addressed the discrepancies/mismatches reported by VS and SAV. If an item was reported when VV and AG filled the table, but not on the day VS or SAV verified, or vice-versa, then that item was marked as unreported.
  - Items in the historical row of the scoring table were marked as reported only if they were reported on all fourteen days during the scoring period.
  - Any item that was not applicable for a state was marked as 'NA'. For example, (i) stratified by districts is not applicable to Chandigarh, as it doesn't have any districts; (ii) for states that reported zero deaths until the end of the scoring period, stratified by age, gender, and comorbidities for deaths were marked as 'NA'. (iii) for a state that doesn't report any data privacy is marked as 'NA'.

The curated data is publicly available at `https://github.com/varun-vasudevan/CDRS-India/tree/master/study1_may`.

[2]Search phrases were of the form "≪statename≫ government covid website" and "≪statename≫ government corona website".

S6 SDG3-II

The indicators used by NITI Aayog to calculate SDG3-II are: (1) Maternal mortality ratio, (2) Proportion of institutional deliveries (%), (3) Under-five mortality rate per 1000 live births, (4) Fully immunised children in the age group 0–5 years (%), (5) Total case notification rate of Tuberculosis per 1 lakh population, (6) HIV Incidence per 1000 uninfected population, (7) Currently married women aged 15–49 years who use any modern method of family planning (%), and (8) Total physicians nurses and midwives per 10000 population.[2,3]. The SDG3-II score for each state/UT is estimated as the average of the normalized values of all the aforementioned indicators. For more details on the SDG India Index and their scoring process, check the NITI Aayog website https://niti.gov.in/sdg-india-index-dashboard-2019-20. Note that we assign the same SDG3-II value to both Ladakh and Jammu & Kashmir.

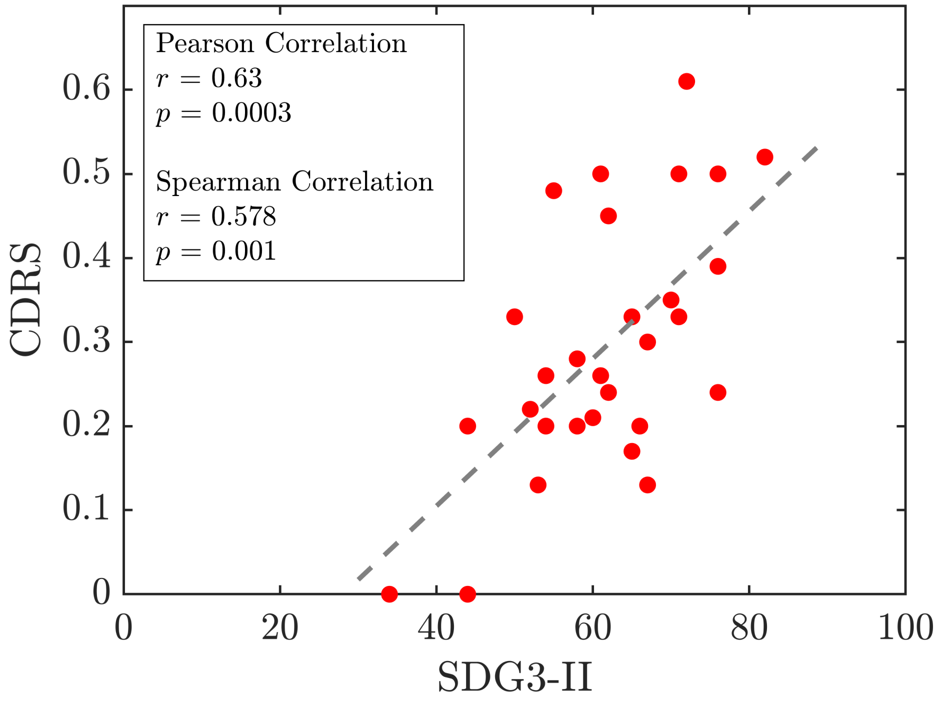

Figure S2: Scatter plot of CDRS versus SDG3-II.

S7 Categorical scores

Below are numerical examples of step-by-step calculation of categorical scores and CDRS for Chandigarh and Gujarat.

Table S1: Step-by-step score calculation for the union territory Chandigarh. Chandigarh doesn't have districts. Therefore, maximum score possible in granularity is 13 and not 18.

**Score**	**Total** $T(c)$	**Max** $M(c)$	**min** $m(c)$	**Normalized** $N(c) = \frac{T(c)}{M(c) - m(c)}$
Availability	4	15	0	$\frac{4}{15-0} = 0.27$
Accessibility	5	12	0	$\frac{5}{12-0} = 0.42$
Granularity	0	13	0	$\frac{0}{13-0} = 0.00$
Privacy	-1	1	-1	$\frac{-1}{1-(-1)} = -0.50$
CDRS	$\frac{\sum_{c \in C} T(c)}{\sum_{c \in C} M(c)} = \frac{4+5+0+(-1)}{15+12+13+1} = \frac{8}{41} = 0.20$			

Table S2: Step-by-step score calculation for the state of Gujarat.

**Score**	**Total** $T(c)$	**Max** $M(c)$	**min** $m(c)$	**Normalized** $N(c) = \frac{T(c)}{M(c) - m(c)}$
Availability	9	15	0	$\frac{9}{15-0} = 0.60$
Accessibility	1	12	0	$\frac{1}{12-0} = 0.08$
Granularity	3	18	0	$\frac{3}{18-0} = 0.17$
Privacy	1	1	-1	$\frac{1}{1-(-1)} = 0.50$
CDRS	$\frac{\sum_{c \in C} T(c)}{\sum_{c \in C} M(c)} = \frac{9+1+3+1}{15+12+18+1} = \frac{8}{46} = 0.30$			

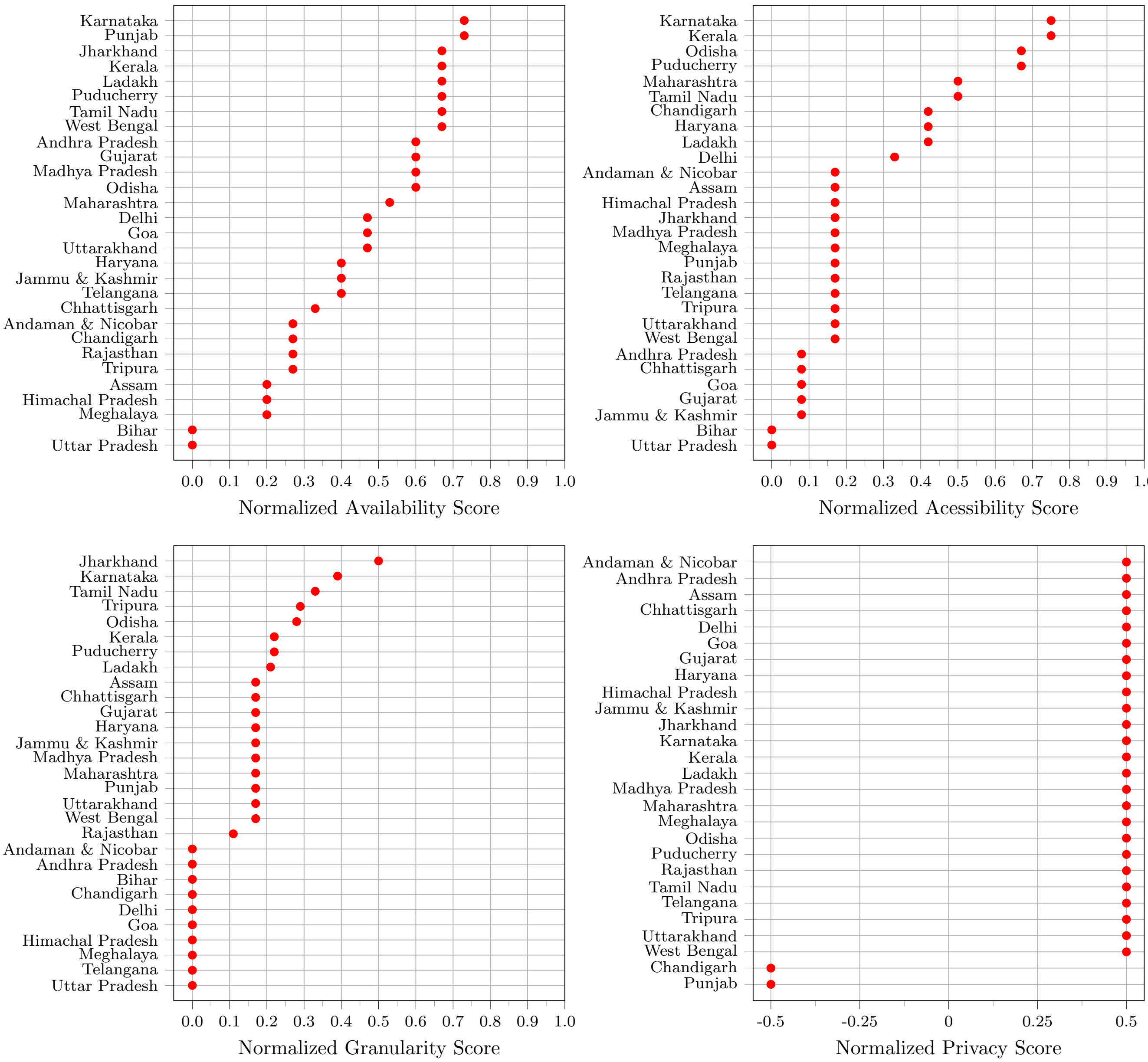

Figure S3: Dot plots showing the normalized availability, accessibility, granularity, and privacy score. Bihar and Uttar Pradesh are not shown in the privacy plot because privacy doesn't apply to them as they don't release any data.

CDRS and the normalized categorical scores for the 29 states and union territories are listed in the table below in the alphabetical order.

Table S3: CDRS and the normalized categorical scores for the states in India. States are listed in the alphabetical order.

	State / Union Territory	Accessibility Score	Availability Score	Granularity Score	Privacy Score	CDRS
1	Andaman and Nicobar Islands	0.17	0.27	0.00	0.50	0.17
2	Andhra Pradesh	0.08	0.60	0.00	0.50	0.24
3	Assam	0.17	0.20	0.17	0.50	0.20
4	Bihar	0.00	0.00	0.00	NA	0.00
5	Chandigarh	0.42	0.27	0.00	-0.50	0.20
6	Chhattisgarh	0.08	0.33	0.17	0.50	0.22
7	Delhi	0.33	0.47	0.00	0.50	0.26
8	Goa	0.08	0.47	0.00	0.50	0.21
9	Gujarat	0.08	0.6	0.17	0.50	0.30
10	Haryana	0.42	0.40	0.17	0.50	0.33
11	Himachal Pradesh	0.17	0.20	0.00	0.50	0.13
12	Jammu and Kashmir	0.08	0.40	0.17	0.50	0.24
13	Jharkhand	0.17	0.67	0.50	0.50	0.48
14	Karnataka	0.75	0.73	0.39	0.50	0.61
15	Kerala	0.75	0.67	0.22	0.50	0.52
16	Ladakh	0.42	0.67	0.21	0.50	0.45
17	Madhya Pradesh	0.17	0.60	0.17	0.50	0.33
18	Maharashtra	0.50	0.53	0.17	0.50	0.39
19	Meghalaya	0.17	0.20	0.00	0.50	0.13
20	Odisha	0.67	0.60	0.28	0.50	0.50
21	Puducherry	0.67	0.67	0.22	0.50	0.50
22	Punjab	0.17	0.73	0.17	-0.50	0.33
23	Rajasthan	0.17	0.27	0.11	0.50	0.20
24	Tamil Nadu	0.50	0.67	0.33	0.50	0.50
25	Telangana	0.17	0.40	0.00	0.50	0.20
26	Tripura	0.17	0.27	0.29	0.50	0.26
27	Uttar Pradesh	0.00	0.00	0.00	NA	0.00
28	Uttarakhand	0.17	0.47	0.17	0.50	0.28
29	West Bengal	0.17	0.67	0.17	0.50	0.35

S8 Screenshot from a bulletin published by Jharkhand

(II) District wise breakup of All COVID-19 Cases								
		Cumulative till Yesterday				Today status		
Sr. No.	District	Total Active Cases	Total Recovered & Discharged	Total Deaths	Total Positive Cases	No. of Positive Cases	Recovered & Discharged	Migrant positive cases districtwise from 2nd May, 2020 onwards
1	Ranchi	21	106	2	**129**	1	0	21
2	Bokaro	7	14	1	**22**	0	0	7
3	Hazaribagh	49	20	0	**69**	3	0	63
4	Dhanbad	26	4	0	**30**	0	0	24
5	Giridih	13	3	1	**17**	0	0	13
6	Simdega	12	2	0	**14**	0	0	12
7	Koderma	15	25	1	**41**	0	0	42
8	Deoghar	0	5	0	**5**	0	0	1
9	Garhwa	11	48	0	**59**	0	0	56
10	Palamu	3	15	0	**18**	0	0	15
11	Jamtara	0	2	0	**2**	0	0	0
12	Godda	0	1	0	**1**	0	0	0
13	Dumka	0	2	0	**2**	0	0	2
14	East Singhbhum	95	1	0	**96**	10	0	87
15	Latehar	6	4	0	**10**	0	0	10
16	Lohardaga	1	2	0	**3**	1	0	3
17	Ramgarh	24	0	0	**24**	1	0	24
18	West Singhbhum	14	1	0	**15**	0	0	14
19	Gumla	21	0	0	**21**	0	0	20
20	Saraikela	4	0	0	**4**	0	0	4
21	Chatra	0	1	0	**1**	0	0	1
22	Pakur	5	0	0	**5**	0	0	5
23	Khunti	5	0	0	**5**	0	0	5
24	Sahebganj	1	0	0	**1**	0	0	1
	**Total**	**333**	**256**	**5**	**594**	**16**	**0**	**430**

(III) Age & Gender wise distribution of All COVID-19 Cases						
Sr. No.	Age Group & Sex	< 10 yrs	11-30 Yrs	31-50 Yrs	51-70 Yrs	> 70 Yrs
1	Male	9	289	189	29	5
2	Female	6	63	11	8	1
	**Total**	**15**	**352**	**200**	**37**	**6**

(IV) Age & Gender wise distribution of Recovered & Discharged COVID-19 Cases						
Sr. No.	Age Group & Sex	< 10 yrs	11-30 Yrs	31-50 Yrs	51-70 Yrs	> 70 Yrs
1	Male	5	104	81	12	3
2	Female	5	33	7	6	0
	**Total**	**10**	**137**	**88**	**18**	**3**

(V) Age & Gender wise distribution of Death (COVID-19 Cases)						
Sr. No.	Age Group & Sex	< 10 yrs	11-30 Yrs	31-50 Yrs	51-70 Yrs	> 70 Yrs
1	Male	0	0	2	1	1
2	Female	0	0	0	1	0
	**Total**	**0**	**0**	**2**	**2**	**1**

Figure S4: This is a screenshot from the bulletin published by the state of Jharkhand on May 23, 2020. It shows how the state publishes granular data.

S9 Additional notes on a few states

- Bihar: The twitter handles https://twitter.com/PIB_Patna/ and https://twitter.com/BiharHealthDept seem to publish some COVID-19 data from Bihar. If we were to consider the data published via twitter, Bihar would get a CDRS between 0 and 0.2. An exact score calculation is not possible without carefully going through all the tweets between 19 May and June 1, 2020.

- Privacy violation in Karnataka: On Mar 25, 2020, a government official from Karnataka released a document containing the details of all persons in a 14-day home quarantine. The document contained residential address (house number, street, district, and pincode) of more than 14k people who were under quarantine. See the original tweet[3] and the comments to the tweet at https://twitter.com/bbmpcomm/status/1242726082102456320?lang=en. The release of personally identifiable information like residential address is a clear violation of privacy. Responses to the tweet are a reflection of public concerns about the compromise in privacy.
  The released document was unavailable during the scoring period of our study — it was probably removed following public concerns. Therefore, we didn't deduct points for privacy for the state of Karnataka. If we were to deduct points, Karnataka's alternate CDRS would be 0.57. The alternate score doesn't change our analysis and conclusions.

[3] These tweets were last accessed on June 28, 2020.

S10 Sources for scoring data

Table S4: Sources for scoring data.

	State / Union Territory	Data Reporting Websites
1	Andaman and Nicobar Islands	https://dhs.andaman.gov.in/
2	Andhra Pradesh	http://hmfw.ap.gov.in/covid_19_dailybulletins.aspx<br>http://hmfw.ap.gov.in/covid_dashboard.aspx
3	Assam	https://covid19.assam.gov.in/
4	Bihar	No sources
5	Chandigarh	http://chdcovid19.in/
6	Chhattisgarh	http://cghealth.nic.in/ehealth/covid19/pages/index.html
7	Delhi	https://delhifightscorona.in/<br>http://web.delhi.gov.in/wps/wcm/connect/doit_health/<br>Health/Home/Covid19/Bulletin+May+2020
8	Goa	https://www.goa.gov.in/covid-19/<br>https://nhm.goa.gov.in/corona-virus-important-links-iec/
9	Gujarat	https://gujcovid19.gujarat.gov.in/
10	Haryana	http://www.nhmharyana.gov.in/page.aspx?id=208<br>https://gisgmda.maps.arcgis.com/apps/dashboards/<br>5cade394ece3496a9e0c4f168f9536a2
11	Himachal Pradesh	http://www.nrhmhp.gov.in/
12	Jammu and Kashmir	https://www.jkinfonews.com/index.aspx
13	Jharkhand	https://www.jharkhand.gov.in/Home/Covid19Dashboard
14	Karnataka	https://covid19.karnataka.gov.in/english
15	Kerala	https://dashboard.kerala.gov.in/index.php
16	Ladakh	http://covid.ladakh.gov.in/
17	Madhya Pradesh	http://mphealthresponse.nhmmp.gov.in/covid/
18	Maharashtra	https://experience.arcgis.com/experience/<br>8167a61f882a4af4b9098e947dfd589f/<br>https://arogya.maharashtra.gov.in/1175/Novel--Corona-<br>Virus
19	Meghalaya	http://meghalayaonline.gov.in/covid/login.htm
20	Odisha	https://statedashboard.odisha.gov.in/<br>https://health.odisha.gov.in/
21	Puducherry	https://covid19dashboard.py.gov.in/<br>https://covid19.py.gov.in/
22	Punjab	https://dronamaps.com/corona.html#/<br>http://pbhealth.gov.in/media-bulletin.htm<br>https://corona.punjab.gov.in/
23	Rajasthan	http://www.rajswasthya.nic.in/
24	Tamil Nadu	https://stopcorona.tn.gov.in/
25	Telangana	http://124.124.103.93/COVID/home.htm
26	Tripura	https://tripura.gov.in/covid-test<br>https://covid19.tripura.gov.in/<br>https://covid19.tripura.gov.in/Visitor/ViewStatus.aspx
27	Uttar Pradesh	No sources
28	Uttarakhand	http://health.uk.gov.in/pages/view/101-covid19-health-<br>bulletin-for-uttarakhand
29	West Bengal	https://www.wbhealth.gov.in/

S11 Total confirmed COVID-19 cases as of May 18, 2020

Table S5: Shows the total number of confirmed COVID-19 cases in 29 states and union territories of India as of May 18, 2020. States are sorted in the order of decreasing number of cases. Source: Ministry of Health and Family Welfare.

	State / Union Territory	Abbreviation	Total Confirmed COVID-19 Cases
1	Maharashtra	MH	33053
2	Gujarat	GJ	11379
3	Tamil Nadu	TN	11224
4	Delhi	DL	10054
5	Rajasthan	RJ	5202
6	Madhya Pradesh	MP	4977
7	Uttar Pradesh	UP	4259
8	West Bengal	WB	2677
9	Andhra Pradesh	AP	2407
10	Punjab	PB	1964
11	Telangana	TS	1551
12	Bihar	BR	1262
13	Jammu and Kashmir	JK	1183
14	Karnataka	KA	1147
15	Haryana	HR	910
16	Odisha	OD	828
17	Kerala	KL	601
18	Jharkhand	JH	223
19	Chandigarh	CH	191
20	Tripura	TR	167
21	Assam	AS	101
22	Uttarakhand	UK	92
23	Chhattisgarh	CG	86
24	Himachal Pradesh	HP	80
25	Ladakh	LA	43
26	Andaman and Nicobar Islands	AN	33
27	Goa	GA	29
28	Meghalaya	ML	13
29	Puducherry	PY	13

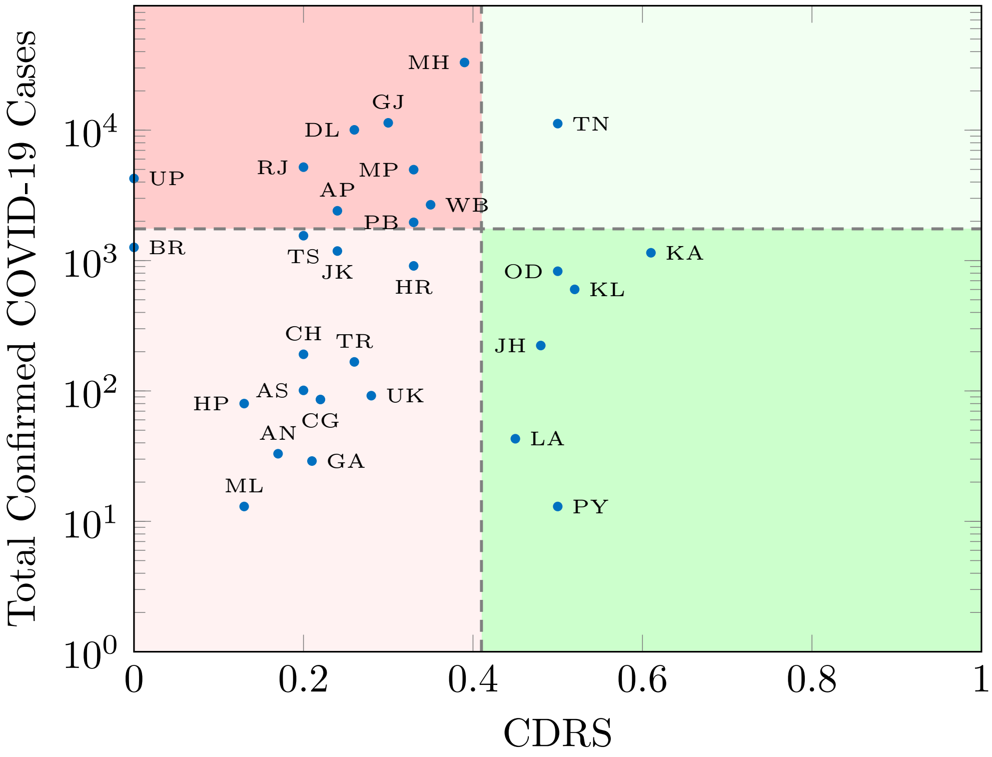

Figure S5: Scatter plot of total confirmed cases versus CDRS. The ten states above the horizontal dashed line contributed to 91% of the total confirmed cases in India as of May 18, 2020. Tamil Nadu is the only state among those 10 with a CDRS in the 75th percentile. The vertical dashed line at 0.41 shows the 75th percentile for CDRS.

References

[1] Jin JM, Bai P, He W, Wu F, Liu XF, Han DM, et al. Gender differences in patients with COVID-19: Focus on severity and mortality. Frontiers in Public Health. 2020;8:152.

[2] UN. About the Sustainable Development Goals;. (accessed June 23, 2020). https://www.un.org/sustainabledevelopment/sustainable-development-goals/.

[3] NITI Aayog. SDG India Index and Dashboard 2019–2020; 2019. (accessed June 25, 2020). https://niti.gov.in/sdg-india-index-dashboard-2019-20.
